# A BODIPY based probe for the reversible “turn on” detection of Au(III) ions

**DOI:** 10.55730/1300-0527.3325

**Published:** 2021-12-18

**Authors:** Muhammed ÜÇÜNCÜ

**Affiliations:** Department of Analytical Chemistry, Faculty of Pharmacy, İzmir Katip Çelebi University, İzmir, Turkey

**Keywords:** BODIPY, 2-aminopyridine, chemosensors, gold(III) ions, “turn on” sensing

## Abstract

A new “turn on” fluorescent probe for the rapid and selective detection of Au^3+^ ions over other metal ions was developed. The probe design was constructed on a BODIPY-2-aminopyridine skeleton showing a weak fluorescence emission signal which increased substantially after the coordination of Au^3+^ ions. The probe displayed remarkable sensing performances such as a low limit of detection (17 nM), a short response time (<1 min), and ability in a wide range of pH’s (6–11). The designed probe was found to have 2:1 coordination stoichiometry according to Job’s plot analysis and most importantly to interact reversibly with Au^3+^ ions.

## 1. Introduction

Gold has attracted great attention in various research fields, including chemistry, electronics, and medicine, due to its unique photochemical and photophysical properties. The outstanding catalytic activity of gold ions (Au^+^ and Au^3+^) allows the progress of many complex organic transformations [1–3]. In addition, its high electrical conductivity, chemical inertness, and malleability increase its significance for electronics applications [4]. In addition to these positive features, the nanoparticle forms of gold have been frequently used in biological systems for imaging applications and drug delivery systems [5,6]. Moreover, gold is present in the structure of many drugs that have been used to treat several significant diseases including rheumatoid arthritis, cancer, AIDS, bronchial asthma, and malaria [7–9]. However, the accumulation of gold ions in living organisms has been previously reported to show high toxicity due to strong interactions with biomolecules (e.g., DNA, enzymes, etc.) [10,11]. Therefore, the development of both sensitive and selective methods to detect gold ions is in high demand.

Recently, fluorometric methods which rely on the changes in fluorescence signal in the presence of target analyte have gained substantial attention for trace metal analysis [12–16]. They offer high sensitivity and selectivity, ease of applicability in solution and biological systems and relatively cheap instrumentation which all together make fluorescent probes excellent alternatives to traditional analytical methods such as inductively coupled plasma atomic emission spectroscopy (ICP-AAS), ICP optical emission spectroscopy (ICP-OES), and ICP mass spectroscopy.

Several fluorescent probes have been specifically designed to detect gold ions by utilizing fluorophores including BODIPY [17,18], Rhodamine [19–20], fluorescein [21–23], coumarin [24], and naphthalimide [25]. Most of these probes were constructed on irreversible reaction-based sensing strategies that are largely based on the alkynophilic character of gold ions [17, 19, 24] or hydrolysis of C = N bond [21, 26, 27]. However, both strategies have some drawbacks, such as the potential cross-reactivity of other metal ions with alkynophilic/Lewis acidic character which might interfere with gold ion sensing. Another approach in probe design is to construct the reversible coordination of gold ions to heteroatom-containing ligands. However, there are only a few studies in the literature that have reported the use of the latter type of probes because of the difficulties in the design and synthesis of single analyte selective ligands [28–30].

Herein, the design, synthesis, and spectral characteristics of a new “turn on” fluorescent molecular probe, BOD-AP, for the selective and sensitive detection of Au^3+^ ions in an aqueous environment have been presented. BODIPY dye was selected as a signal reporter unit due to its exceptional properties such as high fluorescence quantum yield, high extinction coefficient, high photo-stability, and robustness towards chemicals. BODIPY was decorated with a 2-aminopyridine receptor unit bearing heteroatoms that enable the coordination of gold ions. BOD-AP was designed to be a nonfluorescent (“*off mode*”) probe in its initial state due to the photo-induced electron transfer and to convert into fluorescent (“*on mode*”) after the coordination of Au^3+^ ions.

## 2. Materials and methods

### 2.1. General methods

All reagents were purchased from Sigma-Aldrich and used without further purification. ^1^H NMR and ^13^C NMR were measured on a VNMRJ 600 nuclear magnetic resonance spectrometer (Varian Inc., Palo Alto, CA, USA). The mass analysis was conducted with Thermo Q-Exactive Orbitrap device (Thermo Fisher Scientific Inc., Waltham, MA, USA). The melting point was determined by using the Electrothermal Melting Point Apparatus 9200. UV-vis absorption and fluorescence emission spectra were obtained using a Shimadzu 1900i spectrophotometer and Varian Cary Eclipse fluorescence spectrophotometer (Varian Inc.), respectively. Quantum yield measurements were conducted with Hamamatsu Quantaurus-QY Absolute PL quantum yield spectrometer. The pH values were adjusted by using the HI-8014 (HANNA) pH meter.

### 2.2. Fluorescence measurements

The stock solution of BOD-AP (1 mM) was prepared in ethanol and diluted to proper concentrations for each measurement by using different ratios of 0.1 M phosphate buffer/ethanol (total sample volume = 2 mL). The stock solutions of metal ions (20 mM) were prepared by dissolving their nitrate and chloride salts in deionized water. For each measurement, proper volumes from the stock solution of metal ions were added to a probe solution (10 μM, 2 mL) that was contained in a quartz cuvette with 10 mm path length. Upon excitation at 460 nm, the fluorescence emission spectra were collected between 480–700 nm. In all measurements, the slit width for both excitation and emission were kept at 2.5 nm. All measurements were conducted in triplicate at least.

### 2.3. Synthesis of BOD-AP

Meso-chloromethyl-BODIPY (meso-BOD-Cl) was prepared as previously reported [31]. The probe molecule, BOD-AP, was synthesized using a slightly modified version of protocol reported by Xu et al. [32]. To a solution of meso-BOD-Cl (100 mg, 0.34 mmol) in tetrahydrofuran (50 mL) 2-aminopyridine (32 mg, 0.34 mmol), and K_2_CO_3_ (64 mg, 0.4 mmol) were added, and the solution was stirred for 5 h under nitrogen atmosphere at reflux temperature. The progress of the reaction was monitored by TLC and cooled to room temperature when all starting materials consumed. THF was removed under reduced pressure and resulting mixture was extracted with dichloromethane (3 × 100 mL). The collected organic layers were combined and washed with brine, then dried over Na_2_SO_4_, filtered and solvents removed under reduced pressure. The crude mixture purified by silica gel chromatography (Hexane:DCM, 3:1, v/v) and title compound, BOD-AP, was obtained as orange solid (84.3 mg, 70%). Mp: 203–205 °C. ^1^H NMR (600 MHz, CDCl_3_, δ, ppm) δ 8.11 (d, *J* = 4.2 Hz, 1H), 7.38 (t, *J* = 7.2 Hz, 1H), 6.57 (t, *J* = 7.2 Hz, 1H), 6.42 (d, *J* = 8.4 Hz, 1H), 6.24 (s, 1H), 6.10 (s, 1H), 5.18 (s, 1H), 4.79 (d, *J* = 6.6 Hz, 2H), 2.58 (s, 3H), 2.54 (s, 3H), 2.42 (s, 3H), 2.37 (s, 3H). ^13^C NMR (150 MHz, CDCl_3_, δ, ppm) δ 158.6, 155.6, 153.6, 148.2, 142.7 (2×C), 140.5, 137.6, 132.9, 132.2, 122.2, 119.7, 113.4, 107.4, 39.3, 17.6 (2×C), 16.7, 14.7. Calcd for C_19_H_21_BF_2_N_4_: 354.18273 [M]^+^, found: 355.18954 [M + H]^+^.

## 3. Results and discussion

**Scheme 1:**
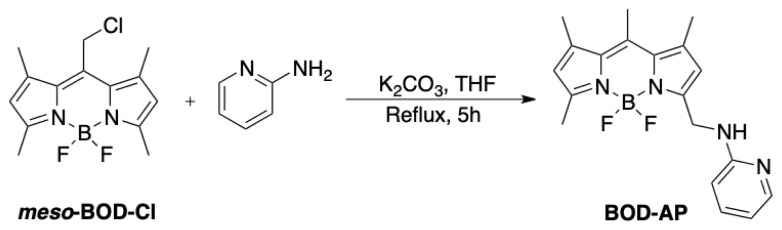
Synthetic route to BOD-AP.

The title compound, BOD-AP was obtained by following the synthetic route outlined in Scheme 1. The meso-BOD-Cl was prepared as previously reported [31] and treated with the commercially available 2-aminopyridine to give BOD-AP as an orange solid with a good reaction yield (70%). The structure of BOD-AP was confirmed by ^1^H and ^13^C NMR spectroscopy and HRMS analysis.

The aromatic protons resonated in the range of 8.11 ppm and 6.10 ppm in the ^1^H NMR spectra proved the presence of a pyridine ring on the probe structure. The NH proton showed a broad singlet peak centred at 5.18 ppm, and the methylene protons resonated at 4.79 ppm which is split into a doublet due to the low exchange rate of proton on the neighbouring nitrogen proton [33, 34]. The obtained spectral profile clearly demonstrated that the nucleophilic substitution of 2-aminopyridine to the BODIPY core took place at the 3-methyl position instead of at the meso position. As reported in the literature, four methyl groups of meso substituted products must have appeared as two singlets (each peak 6H) [32]. However, in obtained ^1^H NMR spectra, the presence of four singlet peaks for methyl groups (each centred at 2.58 ppm, 2.54 ppm, 2.42 ppm, 2.37 ppm) was clear evidence of the position that the nucleophile substituted at 3-position.

**Figure 1:**
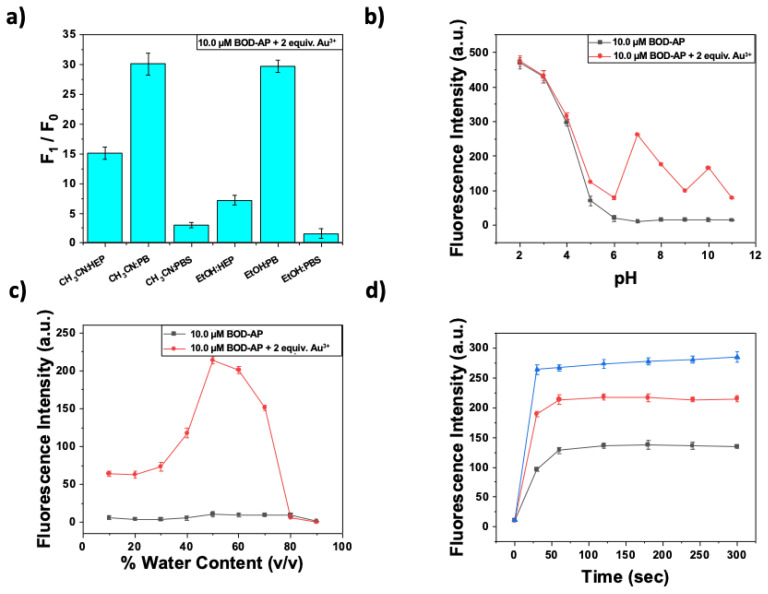
Optimization of sensing conditions BOD-AP for Au^3+^ ion sensing. The effect of a) solvent/buffer type b) water content (%, v/v) c) pH on Au^3+^ sensing event (BOD-AP (10 μM) + Au^3+^ (20 μM)) d) time profile of interaction of BOD-AP (10 μM) with Au^3+^ [5 (■), 10 (**●**) and 20 (▲) μM.] in 0.1 M phosphate buffer/EtOH (pH 7.0, v/v, 1:1) (λ_ex_ = 460 nm, λ_em_ = 511 nm at 25 °C).

The optimization studies of sensing conditions for Au^3+^ ion detection were performed by the investigation of the effect of several parameters including a solvent/buffer type, water ratio, pH, and time. Owing to the hydrophobic core structure of BOD-AP, the addition of an organic cosolvent was required to prevent any aggregation in the solution. In this regard, EtOH-H_2_O (1/1, v/v) and CH_3_CN-H_2_O (1/1, v/v) mixtures buffered at pH 7.0 by HEPES (HEP), phosphate buffer (PB), or PBS were screened. As shown in Figure 1a, the addition of Au^3+^ (2 equiv.) to the BOD-AP solutions (10 μM in EtOH-H_2_O and CH_3_CN-H_2_O mixtures buffered at pH 7.0 by phosphate buffer (0.1 M)) resulted in the same fold increase in the fluorescence signal at 511 nm. Because of its low toxicity and commercial availability, ethanol was selected as a cosolvent to perform further fluorescence measurements. In addition, the effect of the water percentage was investigated where 1:1 (v/v) EtOH/H_2_O ratio was determined as optimal condition (pH 7.0, 0.1 M phosphate buffer) (Figure 1b).

The receptor unit is prone to protonation under acidic conditions (pH 2–5). Therefore, BOD-AP exhibited a strong fluorescence signal (protonation cancels the effect of PET quenching) at this pH range and a negligible increase was observed in the presence of Au^3+^ ions (2 equiv.). At physiological or higher pH values, the receptor unit enables the quench fluorophore over PET mechanism and the addition of Au^3+^ ions, thus resulting in a remarkable increase in fluorescence emission (Figure 1c). After the careful examination of all parameters, the optimal sensing conditions were established as 10 μM BOD-AP in 0.1 M phosphate buffer/EtOH (pH 7.0, 1:1, v/v).

**Figure 2:**
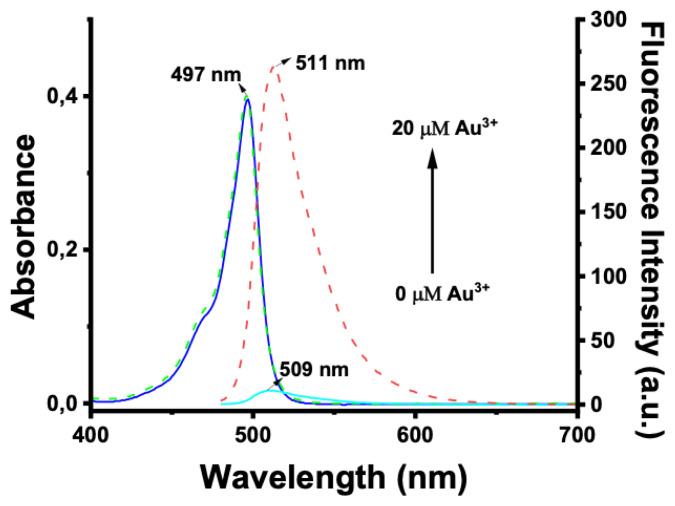
Absorbance spectra of BOD-AP (10 μM) in the absence (blue solid-line) and presence (green dot-line) of 2 equiv. (20 μM) of Au^3+^ and fluorescence spectra of BOD-AP (10 μM) in the absence (cyan solid-line) and presence (red dot-line) of 2 equiv. (20 μM) of Au^3+^ in 0.1 M phosphate buffer/EtOH (pH = 7.0, v/v, 1:1).

**Figure 3:**
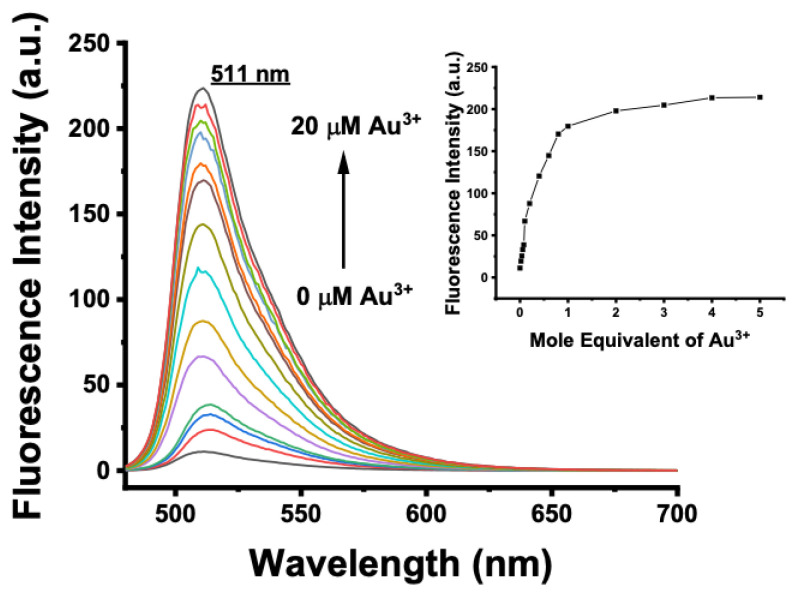
Fluorescence spectra of BOD-AP (10 μM) in the presence of increasing concentrations of Au^3+^ (0–20 μM, 0–2 equiv.) in a 0.1 M phosphate buffer/EtOH (pH = 7.0, 1:1, v/v) (λ_exc_ = 460 nm at 25 °C). Inset: Calibration curve.

The spectral characteristics of BOD-AP were investigated by using UV-vis and fluorescence spectroscopy. BOD-AP has an absorption band at 497 nm which remained unchanged after the addition of Au^3+^ ions (Figure 2). As expected, the probe showed a very weak fluorescence emission (F_F_ = 0.002) centred at 509 nm due to the PET quenching process. Upon the addition of increasing concentrations of Au^3+^ ions (0.2 to 20 μM) a linear increase in fluorescence emission with a very small red shift (λ_em_ = 511 nm) was observed (Figure 3). The saturation point in the fluorescence was achieved by the addition of 2 equiv. of Au^3+^ with a 29-fold signal enhancement (F_F_ = 0.88). The fluorescence titration experiment revealed that the minimum detectable amount of Au^3+^ ions is 17 nM based on the signal-to-noise ratio (S/N = 3) (Figure S1). It is also important to note that the spectral response of the probe towards Au^3+^ ions was extremely fast, reaching maximum levels within only a min (Figure 1d).

**Figure 4:**
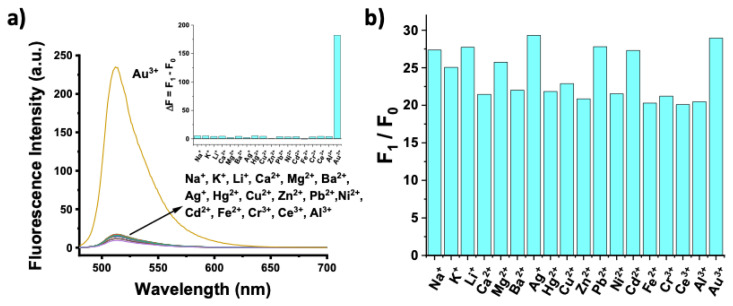
a) Fluorescence spectra of BOD-AP (10 μM), BOD-AP (10 μM) + Au^3+^ (20 μM, 2 equiv.), BOD-AP (10 μM) + other ions (100 μM, 10 equiv.) in 0.1 M phosphate buffer, pH 7.0/EtOH (v/v, 1:1) (λ_ex_: 460 nm, at 25 °C). Inset: Bar graph notation. b) Fluorescence intensities of BOD-AP (10 μM) in the presence of Au^3+^ (20 μM, 2 equiv.) and 10 equiv. of other metal ions in 0.1 M phosphate buffer, pH 7.0/EtOH (v/v, 1:1) (λ_ex_ = 460 nm, λ_em_ = 511 nm at 25 °C).

To evaluate the selectivity profile of the probe, excess amounts of other metal ions (including Na^+^, K^+^, Li^+^, Ca^2+^, Mg^2+^, Ba^2+^, Ag^+^, Hg^2+^, Zn^2+^, Pb^2+^, Ni^2+^, Cd^2+^, Fe^2+^, Cr^3+^, Ce^3+^, and Al^3+^) were added to the BOD-AP solution (10 μM) and then the changes in the fluorescence signals were recorded. BOD-AP did not show any significant response towards the addition of excess amounts (100 μM, 10 equiv.) of any other metal cations (Figure 4a).

Moreover, the possible interference of these species was investigated by the addition of Au^3+^ ions (2 equiv.) to the solution of BOD-AP (10 μM) treated by an excess of other metal cations (100 μM, 10 equiv.). The performed study resulted in only negligible changes in the fluorometric response of the probe to Au^3+^ ions (Figure 4b). These results clearly demonstrate that the probe allows the selective detection of Au^3+^ ions in the presence of any other competitive species. Furthermore, to investigate the applicability of the sensing system in the more complex sensing media, the combination of the potentially interfering metal cations was added into the probe solution. The probe remained silent in the presence of these cations and signal enhancement was only observed upon after the addition of Au^3+^ ions (2 equiv.) (Figure S4).

**Scheme 2:**
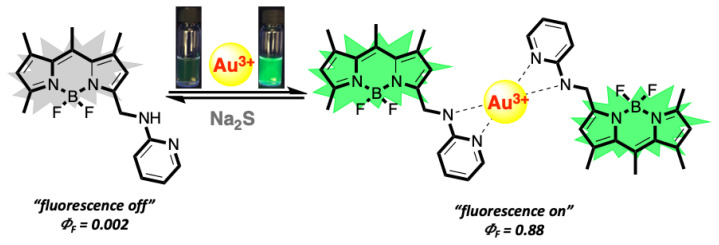
Reversible sensing mechanism of BOD-AP with Au^3+^.

The mechanism and stoichiometry of the sensing event were explored by a reversibility experiment and Job’s plot analysis (Scheme 2). As shown in Figure S2, the treatment of BOD-AP with 2 equiv. of Au^3+^ ions reduced the effect of the PET mechanism, and a strong fluorescence emission band at 511 nm was observed. To reverse this, Na_2_S (2 equiv.) which has a high affinity to Au^3+^ ions was added to the BOD-AP + Au^3+^ solution. The immediate decrease in the fluorescence signal down to its original intensity confirmed the reversibility of the sensing event. In addition, Job’s plot analysis based on fluorescence titration experiment results revealed the 2:1 stoichiometry between BOD-AP and Au^3+^ ions for the complexation process (Figure S3).

In conclusion, synthesis, characterization, and spectral behaviours of a new fluorescent chemosensor that enables the detection of Au^3+^ ions in an aqueous environment were reported. The probe displayed a rapid (<1 min) “turn on” fluorescence response towards Au^3+^ ions with high selectivity and sensitivity (17 nm LoD). In addition, the designed probe showed 2:1 coordination stoichiometry and most importantly reversible interaction with gold ions.

## Supporting Information

### 1. Determination of detection limit

The detection limit of BOD-AP was calculated from the fluorescence titration experiment. Initially, blank solution of BOD-AP (10 μM) was measured by 10 times and the standard deviation of these measurements was determined. Under sensing conditions, a linear relationship between the difference in fluorescence intensity and Au^3+^ ion concentration (0.2–0.8 μM) was obtained (R = 0.9976). The detection limit of BOD-AP was calculated as 17 nM based on the equation;


Detection limit=3σbi/m

where σbi is the standard deviation of blank measurements; m is the slope between intensity versus sample concentration.

Figure S1 Fluorescence changes of BOD-AP (10 μM) upon addition of Au^3+^ (0.2 to 0.8 μM, 0.02 to 0.08 equiv.) in 0.1 M phosphate buffer, pH 7.0/EtOH (v/v, 1:1) (λ_ex_ = 460 nm, λ_em_ = 511 at 25 °C).

### 2. Reversibility of interaction BOD-AP with Au^3+^

Figure S2 Fluorescence spectra of BOD-AP (10 μM) (black), BOD-AP (10 μM) + Au^3+^ (20 μM, 2 equiv.) (red), BOD-AP (10 μM) + Au^3+^ (20 μM, 2 equiv.) + Na_2_S (20 μM, 2 equiv.) (blue) in 0.1 M phosphate buffer, pH 7.0/EtOH (v/v, 1:1) (λ_ex_ = 460 nm, at 25 °C).

### 3. Job’s plot analysis of BOD-AP with Au^3+^

Figure S3 The Job’s plot analysis between BOD-AP and Au^3+^ in 0.1 M phosphate buffer/EtOH (pH 7.0, v/v, 1:1) The total concentration of BOD-AP and Au^3+^ was kept constant at 20 μM (λ_exc_ = 460 nm, λ_em_ = 511 nm at 25 °C).

### 4. Selectivity of BOD-AP towards Au^3+^ in the presence of mixture of metal cations

Figure S4 Fluorescence spectra of BOD-AP (10 μM) (black), BOD-AP (10 μM) + mixture of metal cations (including Na^+^, K^+^, Li^+^, Ca^2+^, Mg^2+^, Ba^2+^, Ag^+^, Hg^2+^, Zn^2+^, Pb^2+^, Ni^2+^, Cd^2+^, Fe^2+^, Cr^3+^, Ce^3+^, and Al^3+^) (100 μM, 10 equiv.) (red), BOD-AP (10 μM) + mixture of metal cations (100 μM, 10 equiv.) + Au^3+^ (20 μM, 2 equiv.) (blue) in 0.1 M phosphate buffer, pH 7.0/EtOH (v/v, 1:1) (λ_ex_ = 460 nm, at 25 °C).

### 5. HRMS spectrum of BOD-AP

Figure S5 HRMS Spectrum of BOD-AP.

### 6. ^1^H and ^13^C NMR of BOD-AP
